# Prognostic analysis of different therapeutic regimens in patients with acute cardiogenic cerebral embolism

**DOI:** 10.1186/s12883-021-02348-9

**Published:** 2021-08-23

**Authors:** Lan Hu, Guangxin Duan, Yuan Xu, Yongjun Cao

**Affiliations:** 1 Department of Neurology, The Second Affiliated Hospital of Soochow University, 1055 Sanxiang Road, Gaoxin District, Room 406, Building 12, Jinyi Garden, Jiangxing West Road, Songling Town, Wujiang District, Jiangsu Province 215004 Suzhou, China; 2 Department of Neurology, The Ninth People’s Hospital of Soochow University, 2666 Ludang Road, Wujiang District, 215200 Suzhou, Jiangsu Province China; 3 Department of Neurology, Nanjing Drum Tower Hospital, 321 Zhongshan Road, Nanjing, Jiangsu Province China

**Keywords:** Anticoagulation, Antiplatelet, Cardiogenic cerebral embolism, Stroke, Thrombolysis

## Abstract

**Background:**

Few studies focused on the functional outcomes of patients at 3 months after receiving intravenous thrombolysis, anticoagulation, or antiplatelet therapy within 4.5 h of onset of the cardiogenic cerebral embolism (CCE) subtype.

**Methods:**

The purpose of this retrospective study was to analyse the clinical data of patients with acute CCE and compare the 3-month functional prognoses of patients after administration of different antithrombotic therapies within 4.5 h of stroke onset. A total of 335 patients with CCE hospitalized in our institution were included in this study. The patients were stratified according to the hyperacute treatment received, and baseline clinical and laboratory data were analysed. A 3-month modified Rankin scale (mRS) score of 0–2 was defined as an excellent functional outcome.

**Results:**

A total of 335 patients were divided into thrombolytic (*n* = 78), anticoagulant (*n* = 88), and antiplatelet therapy groups (*n* = 169). A total of 164 patients had a good prognosis at 3 months (mRS ≤ 2). After adjustments were made for age and National Institute of Health Stroke Scale (NIHSS) score, each group comprised 38 patients, and there were no significant differences in sex composition, complications, lesion characteristics, or Oxfordshire Community Stroke Project (OSCP) classification among the three groups. The plasma D-dimer level (µg/ml) in the thrombolytic group was significantly higher than those in the anticoagulant and antiplatelet groups [3.07 (1.50,5.62), 1.33 (0.95,1.89), 1.61 (0.76,2.96), *P* < 0.001]. After one week of treatment, the reduction in NIHSS in the thrombolytic group was significantly greater than those in the other two groups [3.00 (1.00, 8.00), 1.00 (0.00, 5.00), 1.00 (0.00, 2.00), *P* = 0.025]. A total of 47 patients (41.2 %) had an mRS score of ≤ 2 at 3 months, and 23 patients died (20.2 %). There was no significant difference in the proportion of patients with a good prognosis or the mortality rate among the three groups (*P* = 0.363, *P* = 0.683).

**Conclusions:**

Thrombolytic therapy is effective at improving short-term and 3-month prognoses. Anticoagulant therapy may be a safe and effective treatment option for patients with the cardiac stroke subtype who fail to receive intravenous recombinant tissue plasminogen activator (r-tPA) thrombolysis within 4.5 h in addition to antiplatelet therapy, as recommended by the guidelines.

## Background

Approximately one-fourth of ischaemic strokes are caused by abnormal cardiac structure or function, namely, cardiogenic cerebral embolism (CCE) [1]. The most common cause of CCE is non-valvular atrial fibrillation (NVAF) [2]. Compared with those who have other ischaemic stroke subtypes, patients with CCE have more serious symptoms, poorer prognoses, higher mortality and a higher risk of recurrence or haemorrhagic conversion early after stroke onset [3, 4]. Hyperacute treatment strategies are crucial for improving patient prognosis. For patients whose onset times are within the treatment window, thrombolysis treatment, arterial embolectomy, and bridging therapy are recommended by the American Heart Association/American Stroke Association (AHA/ASA) and European Society of Cardiology (ESC) guidelines [5, 6], and antiplatelet therapy is suggested during the hyperacute stage of CCE [5, 6]; however, the effectiveness of hyperacute anticoagulation remains controversial.

To provide a corresponding clinical reference for CCE subtypes, we retrospectively analysed the clinical characteristics of patients with CCE and compared their 3-month clinical prognoses after receiving different antithrombotic drugs within 4.5 h of stroke onset using data from patients who did not receive either arterial embolectomy or bridging therapy but only drug treatment.

## Methods

### Patient recruitment and criteria

From December 2011 to December 2016, a total of 369 patients with CCE (within 4.5 h of onset) were hospitalized at the Department of Neurology, the Second Affiliated Hospital of Soochow University in Suzhou, China. The follow-up period was 3 months. The study protocol was approved by the Ethics Committee of the Second Affiliated Hospital of Soochow University. Consent to use patient data for clinical analysis was obtained verbally by telephone from the patient or his or her family, and then an informed consent form was signed by the investigators and the patient or his or her family face to face. Patients satisfying the following criteria were enrolled: (1) age > 18 years; (2) average time from onset to admission ≤ 4.5 h; (3) craniocerebral magnetic resonance imaging (MRI) or computed tomography (CT) performed to confirm a diagnosis of new cerebral infarction; (4) diagnostic criteria met for cardiogenic embolism according to Trial of Org 10,172 in the Acute Stroke Treatment (TOAST) classification [7]; and (5) heart disease defined as NVAF with a history of atrial fibrillation of more than 1 month without medication or cardioversion, failure to undergo drug or electrical cardioversion, sinus rhythm that could not be maintained for an extended time, definite dilated heart disease, or rheumatic valvular heart disease. Patients were excluded based on the following criteria: (1) allergy or obvious contraindications to therapeutic drugs, such as recombinant tissue plasminogen activator (r-tPA), warfarin, argatroban, aspirin, clopidogrel, cilostazol, and dabigatran etexilate capsules; (2) a pre-existing haematological disease or an obvious bleeding tendency; (3) severe liver and kidney dysfunction; (4) severe infection; (5) unwillingness to participate and undergo follow-up visits; (6) arterial embolectomy or bridging treatment; (7) gastrointestinal bleeding or gross haematuria; (8) head CT suggesting cerebral haemorrhage at admission; and (9) other stroke subtypes by intracranial and external vascular examinations, imaging or laboratory examinations.

Finally, 335 patients with CCE were stratified into thrombolytic (*n* = 78), anticoagulant (*n* = 88), or antiplatelet (*n* = 169) treatment groups according to the specific drug received within 4.5 h of stroke onset.

### Treatment plan, medication, and monitoring

For thrombolytic therapy, the r-tPA dose was 0.9 mg/kg, with the total dose not exceeding 90 mg. Re-examination by head CT was performed 24 h after administration of thrombolytic treatment and if the patient’s condition changed or before discharge. The antithrombotic regimen at 24 h after thrombolysis was as follows: 1) anticoagulant therapy: (i) 1.25 mg or 1.875 mg warfarin daily with review every 3–5 d using a target international normalized ratio (INR) between 2.0 and 3.0 or (ii) 110 mg dabigatran twice daily, and 2) antiplatelet therapy: (i) 100 mg aspirin daily; (ii) 75 mg clopidogrel daily; (iii) 50 mg cilostazol twice daily; (iv) 100 mg aspirin and 75 mg clopidogrel daily; or (v) 50 mg cilostazol twice daily with 75 mg clopidogrel daily.

For the anticoagulant group, the anticoagulation schemes were as follows: (i) 10 mg argatroban every 4 h for 2 d followed by 10 mg twice daily for 5 d for a total of 7 d or 10 mg argatroban twice daily for 7 d, followed by selection of an individualized antithrombotic regimen; (ii) 1.25 mg or 1.875 mg warfarin daily with review every 3–5 d using a target INR between 2.0 and 3.0; or (iii) 110 mg dabigatran twice daily. Re-examination by head CT was performed if the patient’s condition changed or before discharge; re-examination occurred at least once during hospitalization.

For antiplatelet therapy, the regimens were (i) 100 mg aspirin daily; (ii) 75 mg clopidogrel daily; (iii) 50 mg cilostazol twice daily; (iv) 100 mg aspirin and 75 mg clopidogrel daily; and (v) 50 mg cilostazol twice daily with 75 mg clopidogrel daily. Re-examination head CT was performed if the patient’s condition changed or before discharge; re-examination was performed at least once during hospitalization.

### Patient baseline and clinical characteristics

The following information was collected for each patient: (i) general demographic data (age and sex); (ii) clinical indicators, including aetiology of cerebral embolism (NVAF, rheumatic heart valvular disease, and dilated cardiomyopathy) and concomitant diseases (hypertension, diabetes, prior stroke history, and prior non-cerebrovascular events); (iii) Oxfordshire Community Stroke Project (OCSP) classification [8] with complete anterior circulation (TACI), partial anterior circulation (PACI), posterior circulation (POCI), and lacunar nature (LACI); (iv) laboratory indices, including INR, plasma D-dimer level, C-reactive protein (CRP) level, and serum creatinine (Scr) level; (v) severity, safety, and risk indicators, such as NIHSS at admission and after 1 week of treatment and CHADS2, CHADS2-VASc and HAS-BLED scores; and (vi) prognostic indicators, including the haemorrhagic transformation [9] rate during hospitalization and modified Rankin scale (mRS) score at the 3-month follow-up.

### Statistical methods

All the statistical analyses were performed using SPSS Statistics for Windows version 17.0 (SPSS Inc., Chicago, IL, USA) and R version 4.0.2. Normal distributions are expressed as the mean ± standard deviation (SD). Student’s t-test was used to compare two groups, and one-way ANOVA was used for comparisons among the three groups. Skewed measurements are expressed as the median and interquartile range [*M* (IQR)]. The nonparametric rank-sum test was used for comparisons, post hoc analyses were performed, and pairwise comparisons were performed using the Wilcoxon test with the Bonferroni correction. Count data are expressed as percentages and were compared by means of two-tailed Student’s t-test or Fisher’s exact test. Statistically significant differences were defined as *P*-values < 0.05.

The age and NIHSS indices of the three groups were used as the benchmark to match each group so that the baseline data of the three groups were unified and comparable and bias was eliminated. Propensity score matching (PSM) was used to unify the baseline data of each group. The calliper value selected was 0.02 and matched according to the 1:1 nearest neighbour matching method.

## Results

### Comparison of baseline data and metrics among the CCE-treated subgroups

Our cohort of 335 patients with CCE was divided into three groups according to the therapy adopted within 4.5 h of stroke onset: the thrombolytic (*n* = 78), anticoagulant (*n* = 88), and antiplatelet (*n* = 169) groups. Baseline characteristics are shown in Table 1. The mean ages of the thrombolytic, anticoagulant, and antiplatelet groups were 72.81 ± 8.02, 71.28 ± 9.74, and 76.80 ± 8.41 years, respectively (*P* < 0.001). We found no sex differences among the cohorts, with 39 (50.0 %), 43 (48.9 %), and 77 (45.6 %) males in the thrombolytic, anticoagulant, and antiplatelet groups, respectively (*P* = 0.773).

**Table 1:** Comparison of the baseline data of the enrolled patients receiving different treatment regimens within 4.5 h of stroke onset

Observation index	Thrombolysis group *n* = 78	Anticoagulant group *n* = 88	Antiplatelet group *n* = 169	*P*-value
Age, years, mean ± SD	72.81 ± 8.02	71.28 ± 9.74	76.80 ± 8.41	**< 0.001**
Male, n (%)	39 (50.0)	43 (48.9)	77 (45.6)	0.773
**Aetiology of cerebral embolism**				
Non-valvular atrial fibrillation, n (%)	75 (96.2)	75 (85.2)	161 (95.3)	**0.005**
Rheumatic valvular heart disease, n (%)	3 (3.8)	12 (13.6)	7 (4.1)	**0.008**
Dilated cardiomyopathy, n (%)	0 (0.0)	1 (1.1)	1 (0.6)	0.638
**Concomitant diseases**				
Diabetes, n (%)	13 (16.7)	12 (13.6)	34 (20.1)	0.419
Hypertension, n (%)	69 (88.5)	62 (70.5)	133 (78.7)	**0.018**
Previous history of stroke, n (%)	15 (19.2)	25 (28.4)	49 (29.0)	0.245
Prior non-cerebrovascular events, n (%)	4 (5.1)	5 (5.7)	12 (7.1)	0.809
**Lesion characteristics**				
Single large lesion, n (%)	33 (42.3)	24 (27.3)	53 (31.4)	0.102
Single small lesion, n (%)	8 (10.3)	6 (6.8)	15 (8.9)	0.727
Fusion and multiple lesions, n (%)	27 (34.6)	29 (33.0)	51 (30.2)	0.763
Small, scattered lesions, n (%)	10 (12.8)	29 (33.0)	50 (29.6)	**0.006**
**OCSP classification**				
TACI, n (%)	39 (50.0)	34 (38.6)	64 (37.9)	0.174
PACI, n (%)	27 (34.6)	29 (33.0)	55 (32.5)	0.949
POCI, n (%)	7 (9.0)	11 (12.5)	20 (11.8)	0.743
LACI, n (%)	5 (6.4)	14 (15.9)	30 (17.8)	0.059
**Blood biochemical indices**				
INR (median (IQR))	1.06 (1.01, 1.11)	1.05 (1.00, 1.11)	1.07 (1.01, 1.12)	0.564
Plasma D-dimer, µg/mL, M (IQR)	1.49 (0.98, 5.15)	1.16 (0.80, 1.71)	1.19 (0.75, 2.04)	**0.002**
Scr, µmol/L, M (IQR)	74.00 (60.00, 89.00)	71.00 (60.00, 81.00)	70.00 (57.00, 85.00)	0.466
Hcy, µmol/L, M (IQR)	13.50 (10.30, 17.80)	13.00 (10.60, 15.00)	14.40 (11.10, 18.43)	0.086
CRP, mg/L, M (IQR)	7.05 (6.30, 8.80)	7.00 (6.60, 14.40)	7.00 (6.50, 10.50)	0.406

Abbreviations: *M* Median; *IQR* interquartile range; *OCSP* classification, Oxfordshire Community Stroke Project Classification; *TACI* Total Anterior Cerebral Infarction; *PACI* Partial Anterior Cerebral Infarction; *POCI* Posterior Cerebral Infarction; *LACI* Lacunar Cerebral Infarction; *INR* international normalized ratio; *IQR* interquartile range; *INR* international normalized ratio; *Scr* serum creatinine; *Hcy* homocysteine; *CRP* C-reactive protein

There were 75 (96.2 %), 75 (85.2 %), and 161 (95.3 %) cases of NVAF in the thrombolytic, anticoagulant, and antiplatelet groups, respectively, according to the aetiology of the cerebral embolism (*P* = 0.005). Rheumatic valvular disease ranked second to NVAF. In addition, hypertension was the most common accompanying disease, with 69 (88.5 %), 62 (70.5 %), and 133 (78.7 %) cases in the thrombolytic, anticoagulant, and antiplatelet groups, respectively (*P* = 0.018). We also found no significant differences in OCSP classification among the three groups (TACI: *P* = 0.174; PACI: *P* = 0.949; POCI: *P* = 0.743; and LACI: *P* = 0.059). Regarding the biochemical indices, we found that plasma D-dimer levels were different among the three groups (*P* = 0.002), that the D-dimer polymer level was the highest in the thrombolytic group, and that there were no significant differences in CRP levels among the groups (*P* = 0.406).

### Comparison of rating scales, complications, treatments, and patient prognoses

The median NIHSS scores at admission were 14.00 (10.00,19.00), 9.50 (3.00,13.00), and 8.00 (3.00,15.00) for the thrombolytic, anticoagulant, and antiplatelet groups, respectively (*P* < 0.001); however, we found no significant differences in the NIHSS scores after one week of treatment among the three groups (*P* = 0.059; Table 2). After one week of treatment, the reductions in the NIHSS scores [3.00 (1.00, 9.00), 1.00 (0.00, 3.00), 1.00 (0.00, 2.00), *P* < 0.001] were improved most significantly in the thrombolytic group. Current guidelines [10, 11] recommend using the risk stratification schema congestive heart failure, hypertension, age, diabetes, prior stroke (CHADS2) and congestive heart failure, hypertension, age, diabetes, prior stroke, vascular disease, sex (CHA2DS2-VASc) as secondary prevention for patients with atrial fibrillation. However, the threshold value for predicting the prognosis of CCE is unknown, so we evaluated the CHADS2 and CHA2DS2-VASc scores of the patients in the three groups. We found that the CHADS2 and CHA2DS2-VASc scores were significantly different among the three groups (*P* = 0.005, *P* < 0.001, respectively); similarly, the hypertension, abnormal renal and liver function, stroke, bleeding, labile international normalized ratio, elderly age, drugs or alcohol (HAS-BLED) scores of the three groups were all ≥ 3.

**Table 2:** Prognostic comparison of the different treatment regimens received within 4.5 h of stroke onset among the enrolled patients

Observation index	Total	Thrombolysis group *n* = 78	Anticoagulant group *n* = 88	Antiplatelet group *n* = 169	*P*-value
CHADS2 score, M (IQR)	4.00 (3.00, 4.00)	3.00 (3.00, 4.00)	3.00 (3.00, 4.00)	4.00 (3.00, 4.00)	**0.005**
CHA2DS2-VASc score, M (IQR)	5.00 (4.00, 6.00)	5.00 (4.00, 6.00)	5.00 (4.00, 6.00)	5.00 (5.00, 6.00)	**< 0.001**
HAS-BLED score, M (IQR)	3.00 (3.00, 4.00)	3.00 (3.00, 4.00)	3.00 (3.00, 4.00)	4.00 (3.00, 4.00)	**0.001**
HAS-BLED score ≥ 3 points n (%)	289 (86.27)	66 (84.62)	74 (84.09)	149 (88.17)	0.594
NIHSS score before treatment, M (IQR)	10.00 (4.00, 15.00)	14.00 (10.00, 19.00)	9.50 (3.00, 13.00)	8.00 (3.00, 15.00)	**< 0.001**
NIHSS score after 1-week of treatment, M (IQR)	7.00 (2.00, 14.00)	9.00 (3.00, 17.00)	6.00 (1.25, 12.00)	6.00 (2.00, 14.00)	0.059
Reduction in NIHSS score, M (IQR)	1.00 (0.00, 3.00)	3.00 (1.00, 9.00)	1.00 (0.00, 3.00)	1.00 (0.00, 2.00)	**< 0.001**
Intracranial haemorrhage transformation, n (%)	26 (7.8)	13 (16.7)	4 (4.5)	9 (5.3)	**0.003**
Extracranial haemorrhage transformation, n (%)	4 (1.2)	2 (2.6)	0 (0.0)	2 (1.2)	0.316
Antiplatelet prophylaxis, n (%)	109 (32.5)	10 (12.8)	25 (28.4)	74 (43.8)	< **0.001**
insufficient to regulate secondary prevention, n (%)	167 (49.9)	54 (69.2)	32 (36.4)	81 (47.9)	**< 0.001**
3-month mRS, M (IQR)	3.00 (1.00, 5.00)	3.00 (1.00, 5.00)	2.00 (1.00, 4.00)	3.00 (1.00, 5.00)	0.096
Within-3-month mRS ≤ 2 points n (%)	164 (49.0)	30 (38.5)	53 (60.2)	81 (47.9)	**0.018**
Death within 3 months, n (%)	63 (18.8)	16 (20.5)	11 (12.5)	36 (21.3)	0.209

Abbreviations: *M* median; *IQR* interquartile range; *CHADS2* congestive heart failure, hypertension, age, diabetes, and prior stroke are each assigned 1 point; *CHA2DS2-VASc* congestive heart failure, hypertension, diabetes, 65–74 years of age, female sex, and vascular disease are each assigned 1 point, and prior stroke or transient ischaemic attack and being 75 years of age or older are assigned 2 points; *HAS-BLED* hypertension, abnormal renal/liver function, stroke, bleeding history or predisposition to bleeding, labile international normalized ratio, elderly, and drugs/alcohol concomitantly are each assigned 1 point; *NIHSS* National Institutes of Health Stroke Scale; *TTR* Time Treatment Range; *mRS* modified Rankin scale. Insufficient to regulate secondary prevention, TTR<65% (warfarin) and dabigatran ester were not used in sufficient amounts or over the full course of treatment

Next, we evaluated the haemorrhagic transformation rate of patients with CCE in the acute stage (during hospitalization) and found that it was high. There were 26 cases (7.8 %) of intracranial haemorrhagic transformation, with 13 cases in the thrombolytic group (16.7 %), 4 cases in the anticoagulation group (4.5 %), and 9 cases in the antiplatelet group (5.3 %) (*P* = 0.003); 2 patients in the thrombolytic group had symptomatic intracranial haemorrhage [11], and 1 of these two patients died. There were 4 cases of extracranial haemorrhage among the 335 patients (1.2 %, *P* = 0.316).

A total of 164 patients (49.0 %) in our cohort had a good prognosis at 3 months (mRS ≤ 2), namely, 30 patients (38.5 %) in the thrombolytic group, 53 patients (60.2 %) in the anticoagulant group, and 81 patients (47.9 %) in the antiplatelet group (*P* = 0.018; Table 2).

### Comparison of baseline data and metrics among the CCE-treated subgroups after PSM

The age and NIHSS indices of the three groups were used as the benchmarks to match each group. After PSM, each group had 38 patients. The mean ages of the patients in the thrombolytic, anticoagulant, and antiplatelet groups were 73.53 ± 6.88, 72.05 ± 8.44, and 76.34 ± 9.30, respectively (*P* = 0.076), and the numbers of male patients were 18 (47.4 %), 18 (47.4 %), and 20 (52.6 %), respectively (*P* = 0.869). There were no significant differences in the proportion of NVAF or rheumatic valvular heart disease among the three groups, and there were no patients with dilated cardiomyopathy among the groups. There were no significant differences in the incidences of diabetes mellitus (*P* = 0.074), hypertension (*P* = 0.220), history of stroke (*P* = 0.390), or previous peripheral vascular and cardiovascular events (*P* = 0.365) among the three groups. Similarly, there was no significant difference in the lesion characteristics or OSCP classification among the three groups.

Regarding biochemical indices, the level of the plasma D-dimer polymer was significantly increased in the thrombolytic group, with values of 3.07 (1.50, 5.62), 1.33 (0.95, 1.89), and 1.61 (0.76, 2.96) for the thrombolytic, anticoagulant and antiplatelet groups, respectively (*P* < 0.001). There were no significant differences in the INR (*P* = 0.697) or the levels of Scr (*P* = 0.504), Hcy (*P* = 0.234), or CRP (*P* = 0.523) among the groups (Table 3).

**Table 3:** Post-PSM comparison of the baseline data of the enrolled patients receiving different treatment regimens within 4.5 h of stroke onset

Observation index	Thrombolysis group *n* = 38	Anticoagulant group *n* = 38	Antiplatelet group *n* = 38	*P*-value
Age, years, mean ± SD	73.53 **±** 6.88	72.05 **±** 8.44	76.34 **±** 9.30	0.076
Male, n (%)	18 (47.4)	18 (47.4)	20 (52.6)	0.869
**Aetiology of cerebral embolism**				
Non-valvular atrial fibrillation, n (%)	36 (94.7)	36 (94.7)	36 (94.7)	1.000
Rheumatic valvular heart disease, n (%)	2 (5.3)	2 (5.3)	2 (5.3)	1.000
Dilated cardiomyopathy, n (%)	0 (0.0)	0 (0.0)	0 (0.0)	–
**Concomitant diseases**				
Diabetes, n (%)	4 (10.5)	5 (13.2)	11 (28.9)	0.074
Hypertension, n (%)	33 (86.8)	28 (73.7)	33 (86.8)	0.220
Previous history of stroke, n (%)	1 (2.6)	4 (10.5)	3 (7.9)	0.390
Prior non-cerebrovascular events, n (%)	0 (0.0)	0 (0.0)	1 (2.6)	0.365
**Lesion characteristics**				
Single large lesion, n (%)	15 (39.5)	16 (42.1)	11 (28.9)	0.453
Single small lesion, n (%)	5 (13.2)	2 (5.3)	4 (10.5)	0.619
Fusion and multiple lesions, n (%)	12 (31.6)	10 (26.3)	15 (39.5)	0.467
Small, scattered lesions, n (%)	6 (15.8)	10 (26.3)	8 (21.1)	0.531
**OCSP classification**				
TACI, n (%)	14 (36.8)	19 (50.0)	15 (39.5)	0.470
PACI, n (%)	18 (47.4)	12 (31.6)	12 (31.6)	0.257
POCI, n (%)	2 (5.3)	3 (7.9)	8 (21.1)	0.138
LACI, n (%)	4 (10.5)	4 (10.5)	3 (7.9)	1.000
**Blood biochemical indices**				
INR, M (IQR)	1.08 (1.04, 1.13)	1.06 (1.01, 1.12)	1.09 (1.04, 1.14)	0.697
Plasma D-dimer, µg/mL, M (IQR)	3.07 (1.50, 5.62)	1.33 (0.95, 1.89)	1.61 (0.76, 2.96)	< 0.001^*^
Scr, µmol/L, M (IQR)	75.50 (60.50, 91.00)	71.00 (62.50, 81.00)	75.00 (56.00, 90.50)	0.504
Hcy, µmol/L, M (IQR)	15.66 (12.60, 18.42)	13.70 (11.12, 15.65)	16.60 (11.12, 18.12)	0.234
CRP, mg/L, M (IQR)	7.7 (6.2, 8.6)	7.3 (6.1, 13.0)	7.6 (6.5, 11.0)	0.523

Abbreviations: *M* Median; *IQR* interquartile range; *OCSP* classification, Oxfordshire Community Stroke Project Classification; *TACI* Total Anterior Cerebral Infarction; *PACI* Partial Anterior Cerebral Infarction; *POCI* Posterior Cerebral Infarction; *LACI* Lacunar Cerebral Infarction; *INR* international normalized ratio; *IQR* interquartile range; *INR* international normalized ratio; *Scr* serum creatinine; *Hcy* homocysteine; *CRP* C-reactive protein. –, not applicable. *The level of the thrombolysis group was significantly higher than that of the other two groups after hc post

### Comparison of rating scales, complications, treatments, and patient prognoses after PSM

The CHASD2 scores of the three groups all indicated which patients had a high risk of embolism, and the CHASD2 score was highest in the antiplatelet group (*P* = 0.04); however, there were no significant differences in the CHASD2-VASc scores among the three groups (*P* = 0.142). The HAS-BLED scores indicated patients with a high bleeding risk, but there were no significant differences among the three groups (*P* = 0.073). After PSM, the NIHSS scores of the thrombolytic, anticoagulant, and antiplatelet groups were 11.00 (9.00, 13.75), 13.00 (9.00, 15.00), and 13.00 (5.25, 16.00) (*P* = 0.768), respectively, and the NIHSS reductions after 1 week were 3.00 (1.00, 8.00), 1.00 (0.00, 5.00), and 1.00 (0.00, 2.00), respectively (*P* = 0.025). Among the three groups, the improvement was greatest in the thrombolytic group (*n* = 38, each group). Eleven patients had intracranial haemorrhage conversion, namely, 6 patients in the thrombolytic group, 2 patients in the anticoagulant group, and 3 patients in the antiplatelet group (*P* = 0.281), yet all the patients were asymptomatic. At the same time, there were no extracranial bleeding events in the three groups. There were no significant differences in the proportion of patients with good prognoses at 3 months (mRS ≤ 2) or the number of deaths at 3 months among the three groups (*P* = 0.363; *P* = 0.683) (Table 4).

**Table 4:** Post-PSM prognostic comparison of the different treatment regimens received by the enrolled patients within 4.5 h of stroke onset

Observation index	Total	Thrombolysis group *n* = 38	Anticoagulant group *n* = 38	Antiplatelet group *n* = 38	*P*-value
CHADS2 score, M (IQR)	4.00 (3.00, 4.00)	3.00 (3.00, 4.00)	3.00 (3.00, 4.00)	4.00 (3.00, 4.00)	0.040^*^
CHA2DS2-VASc score, M (IQR)	5.00 (4.00, 6.00)	5.00 (4.00, 5.75)	5.00 (4.00, 6.00)	5.00 (5.00, 6.00)	0.142
HAS-BLED score, M (IQR)	4.00 (3.00, 4.00)	3.00 (3.00, 4.00)	4.00 (3.00, 4.00)	4.00 (3.00, 4.00)	0.073
HAS-BLED score ≥ 3 points, n (%)	102 (89.5)	32 (84.2)	35 (92.1)	35 (92.1)	0.432
NIHSS score before treatment, score, M (IQR)	12.00 (7.25, 15.75)	11.00 (9.00, 13.75)	13.00 (9.00, 15.00)	13.00 (5.25, 16.00)	0.768
NIHSS score after 1-week treatment, M (IQR)	9.00 (2.25, 15.00)	6.50 (2.00, 12.50)	11.50 (4.00, 13.75)	10.50 (3.00, 15.00)	0.442
Reduction in NIHSS score, M (IQR)	1.00 (0.00, 5.00)	3.00 (1.00, 8.00)	1.00 (0.00, 5.00)	1.00 (0.00, 2.00)	0.025^**^
Intracranial haemorrhage transformation, n (%)	11 (9.6)	6 (15.8)	2 (5.3)	3 (7.9)	0.281
Extracranial haemorrhage transformation, n (%)	0 (0.0)	0 (0.0)	0 (0.0)	0 (0.0)	1.000
Antiplatelet prophylaxis, n (%)	103 (90.4)	36 (94.7)	31 (81.6)	36 (94.7)	0.081
Insufficient to regulate secondary prevention, n (%)	101 (88.6)	33 (86.8)	31 (81.6)	37 (97.4)	0.078
3-month mRS, M (IQR)	3.08 (2.13)	2.92 (2.33)	2.97 (2.07)	3.34 (2.00)	0.647
Within 3-month mRS ≤ 2 points, n (%)	47 (41.2)	15 (39.5)	19 (50.0)	13 (34.2)	0.363
Death within 3 months, n (%)	23 (20.2)	9 (23.7)	8 (21.1)	6 (15.8)	0.683

Abbreviations: *M* median; *IQR* interquartile range; *CHADS2* congestive heart failure, hypertension, age, diabetes, prior stroke are each assigned 1 point; *CHA2DS2-VASc* congestive heart failure, hypertension, diabetes, 65–74 years of age, female sex, and vascular disease are each assigned 1 point, and prior stroke or transient ischaemic attack and being 75 years of age or older are assigned 2 points; *HAS-BLED* hypertension, abnormal renal/liver function, stroke, bleeding history or predisposition to bleeding, labile international normalized ratio, elderly, and drugs/alcohol concomitantly are each assigned 1 point; NIHSS, National Institutes of Health Stroke Scale; *TTR* Time Treatment Range; *mRS* modified Rankin scale. Insufficient to regulate secondary prevention, TTR < 65 % (warfarin) and dabigatran ester were not used in sufficient amounts or over the full course of treatment. *hc post: The level of the antiplatelet group was significantly higher than that of the other two groups. **post-test: The level of the thrombolysis group was significantly higher than that of the antiplatelet group

## Discussion

CCE accounts for 14–30 % of all cases of ischaemic stroke [12]. The mortality and disability rate of patients with acute cardiogenic stroke can be as high as 50 % [13]. Currently, r-tPA thrombolysis and antiplatelet therapy are recommended for all types of acute ischaemic stroke (including CCE) [5, 6] within 4.5 h of stroke onset; however, anticoagulation therapy in the hyperacute stage of CCE remains a subject of considerable debate. No previous studies have explored the relationship between different anti-thrombolytic regimens and prognosis within 4.5 h of stroke onset in CCE patients. For the first time, we retrospectively observed the 3-month functional prognoses of 335 CCE patients who received thrombolytic, anticoagulant, or antiplatelet regimens within 4.5 h of stroke onset.

The results of this study showed that among the 335 cases, 38.5 % of patients in the thrombolytic group, 60.2 % of patients in the anticoagulant group, and 47.9 % of patients in the antiplatelet group had a good prognosis (mRS ≤ 2) at 3 months (*P* = 0.018). However, after adjustments for age and NIHSS score, there was no difference in the number of patients with an mRS score of ≤ 2 among the groups at 3 months (*n* = 38). Although the thrombolysis group showed no differences compared to the other two groups in terms of improving disability at 3 months, the reduction in the NIHSS score after 1 week in the thrombolytic group was greater than that in the other two groups (*P* < 0.001), indicating that thrombolysis can significantly improve the short-term prognosis of patients with CCE.

Symptomatic intracranial haemorrhage transformation (sICH) is an important factor affecting the prognosis of patients with CCE. Of the 335 patients, 13 (16.7 %) patients in the thrombolysis group experienced intracranial haemorrhagic transformation during hospitalization, only 2 (2.56 %) of whom had sICH, with 1 patient dying suddenly. There were no cases of sICH in the anticoagulant or antiplatelet groups. After PSM, although there was no significant difference in the conversion rate of intracranial haemorrhage among the three groups (*P* = 0.281), there was still a higher chance of intracranial haemorrhage conversion in the thrombolytic group and 1 patient with sICH in this group. This may also be the reason why the good prognosis of the thrombolytic group at 3 months was not different from that of the other two groups.

In recent years, clinicians have been exploring biological indices as prognostic predictive factors. D-dimer is a specific cross-linked fibrin degradation product, and it is a sensitive biomarker for indicating coagulation and fibrinolytic activation [14]. Studies have shown that D-dimer polymer levels in patients with AF are higher than those in normal controls [15–17] and that D-dimer levels remain an independent predictor of unfavourable functional outcomes and mortality [18]. Elevated plasma D-dimer levels on admission are significantly associated with a poor outcome of acute ischaemic stroke, and the best discriminating factor for poor outcome is a plasma D-dimer level ≥ 3.15 µg/ml [19]. In our study (*n* = 335), the D-dimer level of the thrombolytic group was significantly higher than that of the other two groups (*P* = 0.002). Specifically, after PSM, the D-dimer levels of the thrombolytic, anticoagulant, and antiplatelet groups were 3.07 µg/ml (1.50, 5.62), 1.33 (0.95, 1.89), and 1.61 (0.76, 2.96), respectively (*P* < 0.001). The mean value of the thrombolytic group was 3.07 µg/ml, which was close to the cut-off value for a poor prognosis reported in the previous literature [19].

The D-dimer polymer level of the thrombolytic group was significantly higher than that of the other two groups. This was thought to indicate that the thrombolytic group would have a worse outcome, but the prognosis of the thrombolytic group at 3 months was not inferior to that of the other two groups. This finding conversely indicated that thrombolysis can improve functional outcomes at 3 months, consistent with NINDS [13] and ECASS II [9], and that a higher D-dimer level in the thrombolytic group led to a worse outcome, negatively affecting the advantages of thrombolytic treatment.

Two studies, namely, the European Atrial Fibrillation Trial (EAFT) [20] and Studio Italiano Fibrillation Atriale (SIFA) [21], confirmed the safety of warfarin administration during the acute phase of CCE. Furthermore, four randomized control trials [22–25] showed that the bleeding risk of new non-vitamin K antagonist oral anticoagulants is lower than that of warfarin and that these drugs work quickly, exhibiting an anticoagulant effect that is either superior or comparable to that of warfarin. To date, there have been few small-sample exploratory and observational studies of the use of novel anticoagulants in patients with mild-to-medium acute ischaemic stroke related to NVAF [26–29]. In this retrospective study, the median score of the anticoagulant group was 9.5. We chose to initiate anticoagulation within 4.5 h, which is earlier than the EHRS “1–3–6–12 day” rule [30] and the ASA/AHA “4–14 day” recommendation [5]. Intracranial haemorrhage conversion secondary to ultra-early anticoagulation is the most concerning problem for clinicians; however, in our study, transformation of intracranial haemorrhage occurred in only 4 of 88 patients who received anticoagulant therapy within 4.5 h. After PSM, only 2 of 38 patients experienced transformation of intracranial haemorrhage, and all of these patients exhibited asymptomatic patchy bleeding, not intracranial parenchymal haematoma or non-major bleeding events; furthermore, there were no extracranial bleeding events. In this study, the functional prognosis of the anticoagulant group at 3 months was similar to that of the thrombolytic group. The treatment benefits were speculated to be derived from two aspects: the type of anticoagulant used and the time to anticoagulation initiation. Regarding the anticoagulant type, intravenous argatroban (a direct thrombin inhibitor) is the most common treatment approach, followed by dabigatran anticoagulants instead of controversial heparins, low-molecular-weight heparin and heparinoids. The safety and efficacy of argatroban have been demonstrated in a number of studies [31–35]. New anticoagulants act quickly to reduce the risk of bleeding, which consequently improves prognosis. Regarding the time to anticoagulation initiation, previous studies have reported that blood-brain barrier (BBB) destruction is an independent predictor of haemorrhagic transformation and malignant oedema in patients with acute ischaemic stroke [36, 37]. In this study, anticoagulation was initiated within 4.5 h, when BBB damage was minimal and reversible, so the rate of bleeding conversion was low, the reperfusion injury caused by early anticoagulation was mild, and anticoagulant therapy could reduce small and moving thrombi [38]. These may be important reasons why the good prognosis at 3 months in the anticoagulant group in this study was not inferior to that in the thrombolytic group or the antiplatelet group. Currently, the time between the acute phase and secondary prevention has gradually blurred. Given the above findings, we recommend that for some patients with CCE who fail to receive intravenous thrombolytic therapy within 4.5 h, clinicians may consider new novel anticoagulants an effective option. Our future research directions aim to observe the changes in the dynamic BBB and its relationship with prognosis in patients with CCE treated with novel anticoagulants at the hyperacute stage.

At present, the guidelines for the acute phase of CCE anticoagulation with respect to starting time are still inconclusive, and exploratory research is encouraged. This study found that the risk of anticoagulation bleeding in patients with CCE within 4.5 h is not high, which may remind us that in addition to the guideline-recommended anticoagulation time frame (1-3-6-12 days, 4–14 days), 4.5 h may be another time point to start anticoagulation. It must be mentioned that our study is a single-centre study with a small sample size, and more studies are needed to confirm the findings.

Antiplatelet therapy is an important treatment option for CCE patients in the acute phase. The results of randomized controlled studies, namely, IST [39], CAST [40], CHANCE [41], and POINT [42], established the role of aspirin in the treatment of ischaemic stroke during the acute phase. In our study, of 335 patients, 169 chose antiplatelet therapy within 4.5 h, and 9 underwent intracranial haemorrhage transformation (5.3 %); all were asymptomatic, and 2 experienced extracranial haemorrhage (1.2 %). After adjustments were made for age and NIHSS score, there was no significant difference in the incidences of intracranial or extracranial haemorrhage between the antiplatelet group and the other two groups. In this study, initiating antiplatelet therapy within 4.5 h did not lead to a better outcome in terms of safety than thrombolysis and anticoagulation. Although the incidence of bleeding is not high, the risk of bleeding due to antiplatelet therapy should not be ignored by clinicians or patients. According to the results of this study, there was no significant difference in 3-month functional outcomes between the antiplatelet therapy group and the other two groups.

The data in this study showed that the proportion of patients with anticoagulant prophylaxis for CCE was very low from 2011 to 2016, which is very frustrating and concerning. With the popularity of new anticoagulants and the development of anticoagulant prophylactic treatment, the proportion of CCE patients choosing anticoagulants for secondary prevention is gradually increasing.

Our study has a number of limitations. It is a single-centre, retrospective study with a modest sample size, but it is a study of real-world CCE. Future multicentre, prospective, randomized controlled trials with large patient cohorts are warranted.

## Conclusions

Thrombolytic therapy should be the first-line therapy for CCE patients within 4.5 h of stroke onset. For patients who fail to receive intravenous r-tPA thrombolysis within this time window, hyperacute anticoagulant therapy may be an available option as well as antiplatelet therapy.

## Data Availability

All the summarized and analysed data during this study are included in this published article; the original data in this study are available from the corresponding author upon reasonable request.

## Abbreviations

CCE: Cardiogenic cerebral embolism
CHA2DS2-VASc: Congestive heart failure, hypertension, age, diabetes, prior stroke, vascular disease, sex
CHADS2: Congestive heart failure, hypertension, age, diabetes, prior stroke
CRP: C-reactive protein
CT: Computed tomography
EAFT: European Atrial Fibrillation Trial
HAS-BLED: Hypertension, abnormal renal and liver function, stroke, bleeding, labile international normalized ratio, elderly age, drugs or alcohol
Hcy: Homocysteine
INR: International normalized ratio
IQR: Interquartile range
LACI: Lacunar cerebral infarction
M: Median
MRI: Magnetic resonance imaging
mRS: Modified Rankin scale
NIHSS: National Institutes of Health Stroke Scale
NVAF: Non-valvular atrial fibrillation
OCSP: Oxfordshire Community Stroke Project
PACI: Partial anterior cerebral infarction
POCI: Posterior cerebral infarction
r-tPA: Recombinant tissue plasminogen activator
Scr: Serum creatinine
SIFA: Studio Italiano Fibrillation Atriale
TACI: Total anterior cerebral infarction
TTR: Time treatment range
